# The Implications of Post-coital Intravaginal Cleansing for the Introduction of Vaginal Microbicides in South Africa

**DOI:** 10.1007/s10461-013-0676-9

**Published:** 2013-12-13

**Authors:** Mitzy Gafos, Robert Pool, Misiwe Adelaide Mzimela, Hlengiwe Beauty Ndlovu, Sheena McCormack, Jonathan Elford, the MDP team

**Affiliations:** 1 Africa Centre for Health and Population Studies, University of KwaZulu-Natal, Mtubatuba, South Africa; 2 Division of Health Services Research and Management, City University London, London, UK; 3 Institute of Clinical Trials & Methodology, Medical Research Council Clinical Trials Unit at UCL, London, UK; 4 Barcelona Centre for International Health Research, University of Barcelona, Barcelona, Spain; 5 Centre for Global Health and Inequality, University of Amsterdam, Amsterdam, The Netherlands; 6 Educational Professional Practice Unit, University of Zululand, KwaDlangezwa, South Africa; 7 Department of Nursing Science, University of Zululand, KwaDlangezwa, South Africa

**Keywords:** Microbicides, Acceptability, Adherence, Post-coital intravaginal cleansing, South Africa

## Abstract

Post-coital intravaginal cleansing (IVC) could counteract the protective effect of a vaginal microbicide. IVC less than 1 h after sex is discouraged in most microbicide trials. During a microbicide trial in KwaZulu-Natal, we collected quantitative data on post-coital IVC. We discussed IVC during in-depth-interviews (IDIs) and focus-group discussions (FGDs) with women enrolled in the trial, and during FGDs with community members. Nearly one-third (336/1,143) of women reported IVC less than an hour after sex. In multivariate analysis, post-coital IVC was associated with younger age, larger household size, greater sexual activity, consistent gel use, and clinic of enrolment. During IDIs and FGDs, respondents described post-coital IVC as a common hygiene practice motivated by the need to remove semen, vaginal fluids and sweat, although this practice may be amenable to change in the context of microbicide use. We need to consider strategies for influencing post-coital IVC practices in future microbicide trials and delivery programmes.

## Introduction

In 2010, the CAPRISA 004 trial provided proof for the concept of vaginal microbicides [1]. The trial demonstrated a 39 % reduction in the risk of HIV acquisition among women assigned to use tenofovir microbicide gel before and after sex when compared to placebo gel. If the FACTS 001 confirmatory trial supports these results [2], vaginal microbicides may well be introduced as an additional HIV prevention option for women in South Africa. A number of behavioural factors are likely to influence the effectiveness of microbicides. The primary factor is whether women use microbicides *when* expected [3]. However, an additional factor is whether women use microbicides *as* expected. In microbicide trials to date, women have usually been advised to insert a microbicide gel either before sex, or before and after sex, and to refrain from intravaginal cleansing for at least an hour after sex. This is due to concerns that post-coital intravaginal cleansing could remove the microbicide too soon after male ejaculation and counteract the protective effect of the microbicide [4].

The World Health Organisation define intravaginal cleansing as internal cleansing or washing inside the vagina which includes wiping the internal genitalia with fingers and other substances (e.g., cotton, cloths, paper) for the purpose of removing fluids. It also includes douching, which is the pressurised shooting or pumping of water or solution (including douching gel) into the vagina [5]. There has been extensive research into women’s intravaginal cleansing practices in sub-Saharan Africa. In South Africa, studies in the general population have found that the percentage of women reporting intravaginal cleansing varies considerably, from 13 % in the Western Cape to 87 % in Gauteng [5–14], although the prevalence was higher among commercial sex workers [15–18]. Intravaginal cleansing is practiced for a variety of reasons such as maintaining vaginal hygiene and health, treating infections, and preparing for sex [5]. The reasons for the practice influence the timing, frequency and type of cleansing performed [5, 11, 19, 20]. The main concern for microbicide effectiveness relates to post-coital intravaginal cleansing within the first hour after sex.

Only two studies have reported on *post-coital* intravaginal cleansing in South Africa; one study reported a prevalence of 9 % and the other 19 % [11, 21]. Although neither of these studies reported on how long after sex intravaginal cleansing was performed, one study in Tanzania found that half the women who intravaginally cleansed after sex, did so within 2 h [22]. While a number of microbicide trials have reported the prevalence of intravaginal cleansing at baseline, ranging from 25 to 100 % [23–27], only two specifically measured intravaginal cleansing in relation to sexual activity and only one of these reported on it during follow-up. In the HPTN 035 study, at baseline around a quarter of women reported intravaginal cleansing before sex and a similar percentage after sex [28]. In the Cellulose Sulphate trial in Nigeria, nearly three quarters of women reported intravaginal cleansing after sex at baseline but this fell to 6 % during follow-up [29]. There are still gaps in our understanding of post-coital intravaginal cleansing practices and the use of vaginal microbicides [30].

In this paper, we use quantitative and qualitative data collected as part of the Microbicides Development Programme (MDP) 301 clinical trial in KwaZulu-Natal, South Africa to examine post-coital intravaginal cleansing during a microbicide trial. Using quantitative data, we investigate patterns of post-coital intravaginal cleansing during the course of the trial and characterize women who practice intravaginal cleansing less than an hour after sex. Using qualitative data, we examine socio-cultural norms relating to intravaginal cleansing and explore intravaginal cleansing practices among women using microbicide gels. We then consider the implications of post-coital intravaginal cleansing practices for the introduction of microbicides in a rural part of KwaZulu-Natal, South Africa.

## Methods

### Quantitative Methods

#### Cohort

The Africa Centre for Health and Population Studies [31] was one of six research centres conducting the MDP 301 randomised, double-blind, placebo-controlled, phase III clinical trial that evaluated the safety and efficacy of PRO2000 microbicide gel in the prevention of vaginally-acquired HIV infection [32–34]. In total, 1,177 women enrolled in the Africa Centre MDP 301 trial in KwaZulu-Natal from March 2006 to August 2008 with follow-up visits continuing until August 2009. We excluded from the analyses 34 women who did not provide data on intravaginal cleansing, including a total of 1,143 women who provided data on intravaginal cleansing.

We enrolled and followed up women at three research clinics: clinic one was located in a township, clinic two was located in a small town, and clinic three was located in a rural area under tribal authority. All three clinics recruited women from rural areas in addition to the immediate locale of the clinic. At enrolment, women were randomized to use 2 % PRO2000, 0.5 % PRO2000 or placebo gel until February 2008 when evaluation of 2 % PRO2000 was discontinued due to futility, after which time women were randomised to use 0.5 % PRO2000 or placebo gel [35]. We counselled women to insert a pre-filled applicator of gel no more than 1 h before each sex act, and to refrain from intravaginal cleansing for at least 1 h after sex.

#### Dependent Variable

Counsellors administered sexual behaviour questionnaires at clinic visits 4, 24, 40 and 52 weeks after enrolment. We collected data about each sex act in the last week, or the last 4 weeks if a woman had not had sex in the last week. For each sex act, we asked women the following question: “Did you clean inside your vagina after sex?” Staff clarified that intravaginal cleansing included any form of cleansing inside the vagina including the use of water, fingers or a dry cloth. Based on the investigators’ brochure, participants were advised not to intravaginally cleanse for up to an hour after sex as this may counteract the protective effect of the microbicide. As such, we asked women who reported cleansing inside their vagina after sex, how long after sex they had cleansed. Although we were interested in capturing data on post-coital cleansing within 1 h of sex, we asked about additional time periods to further understand local practices. We recorded responses as either less than 1 h, between 1 and 2 h, or more than 2 h after sex. The outcome measure for this analysis is cleansing inside the vagina less than 1 h after sex at any time during the trial.

#### Independent Variables

Baseline independent variables considered in this analysis included age, highest educational level attained, employment status, area of residency, religion, use of reliable contraception (injectable, oral pill, sterilised), relationship to the head of the household (as a proxy for cohabitation if the woman reported her partner as the household head, although cohabitation cannot be ruled out in other head of household relationships), and household size (measured using the number of adults who usually sleep in the household divided by the number of rooms usually used for sleeping). We also considered longitudinal behavioural variables including consistent gel and consistent condom use throughout the trial, average sexual frequency, discussing gel use with a partner, sex during menstruation or multiple partners at any time during the 12 month follow up period. We assessed associations with clinical outcomes based on the results of HIV, pregnancy, gonorrhoea, chlamydia, Trichomonas vaginalis and syphilis testing conducted during the trial. We also considered clinic of enrolment and gel randomisation group.

#### Quantitative Analysis

We compared women who reported intravaginal cleansing (IVC) less than 1 h after sex at some time during the trial to those who did not. We also considered changes over time in intravaginal cleansing less than 1 h after sex by comparing the proportions of women who intravaginally cleansed in the first 6 months of the trial to the proportion in the last 6 months of the trial. We assessed univariate associations with intravaginal cleansing using the Pearson Chi^2^ test. We tested the contribution to the multivariable model of each variable that was significant in univariate analysis at the 0.10 level using likelihood ratio tests (LRT) [36]. We assessed multivariate associations at the 0.05 level, after controlling for potential confounding factors, through multiple logistic regression analyses. Data were analysed using Stata 10 (StataCorp, College Station, Texas, USA).

### Qualitative Methods

#### Cohort

At enrolment, 101 trial participants were randomly selected to participate in in-depth interviews (IDIs). Each woman randomly selected for IDIs was invited to interview three times during the trial, at 4, 24 and 52 weeks after enrolment, in order to capture women’s experiences at the beginning, middle and end of follow-up. Of these, 12 women refused to participate mainly due to the time commitment, one woman withdrew from the trial before the first interview, and four were never available for interview. Consequently, a total of 84 women participated in interviews. Ten women were interviewed once, 18 were interviewed twice, and 56 were interviewed three times for a total of 214 interviews with these 84 women. The interview guide included the following topics: study acceptability and comprehension, gel acceptability, gel and condom use, partnership types and involvement in gel use, risk perception, sexual practices, and vaginal practices including cleansing and insertion. Women who reported washing inside their vagina after sex, were asked how long after sex they had washed. Data included in this paper relate to washing within approximately an hour after sex. We invited trial participants not randomly selected for IDIs, to participate in focus group discussions (FGDs) on an ad hoc basis. We advertised FGDs at the MDP clinics and stratified them by age and clinic of enrolment. During the course of the trial 10 standard FGDs were conducted with an average of nine women per group, ranging from five to 20 women. A total of 77 trial participants took part in the 10 FGDs. The FGD guide for trial participants included the same topics as the trial IDI guide.

We also advertised FGDs at community events and conducted them with women and men who were resident in the trial catchment area but not enrolled in the trial. Community FGDs were stratified by sex, age and area of residence. During the course of the trial 17 standard FGDs were conducted with community members with an average of nine women or men per group ranging from 5–13. In total six FGDs were conducted with 54 women and 11 FGDs were conducted with 103 men. The topics discussed in the community FGDs were the MDP trial, theoretical gel acceptability, partner involvement in gel use, sexual practices and vaginal practices including cleansing and insertion.

Demographics of the IDI and FGD respondents are shown in Tables 1 and 2. In the qualitative results section, we refer to the trial participants as well as women and men from the community who took part in the IDIs and FGDs, as respondents to avoid confusion with specific participants enrolled in the trial.

**Table 1:** Demographics of IDI respondents

	Trial IDIs
No of trial participants	84
No of IDIs	214
Mean age (range)	34 (19–64)
Employed	19 %
Married^a^	24 %
Secondary school education or above	54 %
Rural residency^b^	77 %
Partner as head of household	39 %
Consistent gel user	57 %
Consistent condom user	40 %
Clinic of recruitment	
Clinic 1	32 %
Clinic 2	39 %
Clinic 3	29 %

^a^Marital status was ascertained from the IDI narratives

^b^Residency missing for one women; one urban resident, remainder peri-urban

**Table 2:** Demographics of FGD respondents

	Trial FGDs	Community FGDs (female)	Community FGDs (male)
No of people	77	54	103
No of FGDs	10	6	11
Mean age (range)	36 (19–65)	37 (21–63)	30 (17–67)
Employed^a^	21 %	13 %	5 %
Married^a^	27 %	41 %	14 %

^a^Employment and marital status were collected in all but two of the trial FGDs

#### Qualitative Analysis

We conducted IDIs and FGDs in isiZulu. They were audio recorded, transcribed, translated into English, and imported into NVivo 2, later NVivo 8, for coding (NVivo qualitative data analysis software; QSR International Pty Ltd. Version 2, 2002; Version 8, 2008). The majority of transcripts included both the isiZulu transcription and English translation, although only the English translation was available for 51 IDIs and eight FGDs when direct audio-translation was used with additional quality control of the translated text. Coding was conducted in English. The credibility and trustworthiness of interpretations were considered throughout the trial by presenting results of sub-analyses to local staff and members of the community and participant advisory boards.

We conducted thematic analysis in two stages. Firstly, we analysed the 17 community FGDs and 10 trial participant FGDs, coding all text that addressed issues relating to intravaginal cleansing immediately after sex. Two main themes emerged from the data: classification of, and motivation for intravaginal cleansing. Secondly, we analysed data from the 214 in-depth interviews, again coding all text that addressed issues relating to intravaginal cleansing immediately after sex. Three main themes emerged from the data: intravaginal cleansing practices generally, intravaginal cleansing in relation to gel use, and intravaginal cleansing in relation to sex during menstruation.

Participants provided written informed consent for trial enrolment. In addition, trial participants and community members provided written informed consent for participation in IDIs and FGDs. The University of KwaZulu-Natal Biomedical Ethics Committee (T111/05) and the South African Medicine Controls Council (N2/19/8/2) reviewed and approved the trial protocol.

## Results

### Quantitative Analysis

Of the 1,143 women included in the analysis, 336 (29 %) reported cleansing inside their vagina *less than 1* *h* after sex at some point during the trial [464 (41 %) reported cleansing inside their vagina *at any time* after sex]. Women who intravaginally cleansed less than 1 h after sex were younger than women who did not (mean age 33 vs. 35 years, *t* test *p* value 0.020) and there was a linear correlation with age (OR 0.99; *p*-value 0.021).

As shown in Table 3, post-coital intravaginal cleansing less than 1 h after sex was associated at the 10 % level with age group, living in a rural area, living in a large household, consistent gel use, consistent condom use, discussing gel with a partner, having multiple sex partners, enrolling in the trial in clinics 2 or 3, and having more frequent sex. In terms of sexual frequency, women reported a mean of 4.8 sex acts a week on average during the trial (range 1–15.5, SD 2.09) and there was a linear correlation between post-coital intravaginal cleansing and sexual frequency after adjusting for age (AOR 1.11; *p*-value 0.001). Intravaginal cleansing practices did not differ by gel randomisation group (*p* = 0.189). We excluded consistency of condom use from the multivariate model, as this variable did not contribute to the model in likelihood ratio tests (LRT *p*-value 0.526).

**Table 3:** Characteristics of women who did and did not intravaginally cleanse less than 1 h after sex

Characteristics	Total sample *N* (col %)	Did not intravaginally cleanse less than 1 h after sex *n* (row %)	Intravaginally cleansed less than 1 h after sex *n* (row %)	Chi2 *p*-value
	1,143 (100 %)	807 (71 %)	336 (29 %)	
Age group				0.084
18–24	326 (29 %)	216 (66 %)	110 (34 %)	
25–34	243 (21 %)	175 (72 %)	68 (28 %)	
35–44	274 (24 %)	190 (69 %)	84 (31 %)	
45+	300 (26 %)	226 (75 %)	74 (25 %)	
Educational level				0.453
Primary or lower	557 (49 %)	398 (71 %)	159 (29 %)	
Incomplete secondary	367 (32 %)	262 (71 %)	105 (29 %)	
Complete secondary	219 (19 %)	147 (67 %)	72 (33 %)	
Employment status				0.644
Unemployed	948 (83 %)	672 (71 %)	276 (29 %)	
Employed	195 (17 %)	135 (69 %)	60 (31 %)	
Area of residency				0.008
Rural	899 (79 %)	618 (69 %)	281 (31 %)	
Peri-urban/urban	244 (21 %)	189 (77 %)	55 (23 %)	
Religion				0.312
Christian	250 (22 %)	181 (72 %)	69 (28 %)	
Zionist	528 (46 %)	369 (70 %)	159 (30 %)	
Shembe	279 (24 %)	190 (68 %)	89 (32 %)	
None/other	86 (8 %)	67 (78 %)	19 (22 %)	
Household size				0.008
3 people or more	632 (55 %)	426 (67 %)	206 (33 %)	
1–2 people per room	511 (45 %)	381 (75 %)	130 (25 %)	
Relationship to household head				0.351
Partner	484 (42 %)	351 (73 %)	133 (27 %)	
Parent	394 (35 %)	265 (67 %)	129 (33 %)	
Self	123 (11 %)	88 (72 %)	35 (28 %)	
Other	142 (12 %)	103 (73 %)	39 (27 %)	
Contraceptive use				0.461
No	567 (50 %)	406 (72 %)	161 (28 %)	
Yes	576 (50 %)	401 (70 %)	175 (30 %)	
Gel use				0.031
Sometimes/never	436 (38 %)	324 (74 %)	112 (26 %)	
Always	707 (62 %)	483 (68 %)	224 (32 %)	
Condom use				0.083
Always	494 (43 %)	340 (69 %)	154 (31 %)	
Sometimes	426 (37 %)	296 (69 %)	130 (31 %)	
Never	223 (20 %)	171 (77 %)	52 (23 %)	
Discuss gel with partner				0.042
Yes	939 (82 %)	651 (69 %)	288 (31 %)	
No	204 (18 %)	156 (76 %)	48 (24 %)	
Average no. of sex acts				<0.001
1 to 5 acts	872 (76 %)	646 (74 %)	226 (26 %)	
6 + acts	271 (24 %)	161 (59 %)	110 (41 %)	
Multiple partners				0.065
No	1,022 (89 %)	717 (70 %)	305 (30 %)	
Yes	14 (1 %)	7 (50 %)	7 (50 %)	
Missing	107 (9 %)	83 (78 %)	24 (22 %)	
Clinic of enrolment				<0.001
Clinic 1	438 (38 %)	369 (84 %)	69 (16 %)	
Clinic 2	369 (32 %)	239 (65 %)	130 (35 %)	
Clinic 3	336 (30 %)	199 (59 %)	137 (41 %)	

Although we did not collect data on marital status or cohabitation, the vast majority (99 %) of women reported being in stable, long-term relationships. As a proxy for cohabitation, we compared intravaginal cleansing practices based on a woman’s relationship to the head of the household and found no statistically significant differences (*p* = 0.351).

We compared post-coital intravaginal cleansing among women who had HIV seroconverted, become pregnant, or been diagnosed with gonorrhoea, chlamydia, Trichomonas vaginalis or syphilis during the trial, to those who had not (data not presented due to space but available from authors on request). Intravaginal cleansing was not associated with receiving a diagnosis of HIV, chlamydia, Trichomonas vaginalis or syphilis. A higher proportion of women diagnosed with gonorrhoea reported post-coital intravaginal cleansing (42 % *p* = 0.021), while a lower proportion of women who became pregnant during the course of the study reported post-coital intravaginal cleansing (19 % *p* = 0.041). However, neither gonorrhoea (LRT *p*-value 0.113) nor pregnancy (LRT *p*-value 0.153) contributed to the model in likelihood ratio tests so they were not included in the multivariate model.

We did not collect data relating specifically to post-coital intravaginal cleansing around the time of menstruation. Seventy (6 %) women reported having sex during menstruation at some time during the trial, but this was not associated with post-coital intravaginal cleansing (*p* = 0.700). Eighty-seven percent (61/70) of these women reported typically using gel when having sex during menstruation.

Table 4 presents the output from the final multivariate model. In the multivariate model, women who intravaginally cleansed less than 1 h after sex were more likely to live in larger households, consistently use gel, report greater sexual activity and were more likely to have enrolled at the clinics in the town (clinic 2) or the tribal authority area (clinic 3). Women aged 45 years or older were less likely to cleanse intravaginally after sex.

**Table 4:** Multivariate model comparing women who did and did not intravaginally cleanse less than 1 h after sex

	Adjusted OR	95 % CI	*P* value
Age group			
18–24	1.00		
25–34	0.74	0.50, 1.09	0.124
35–44	0.75	0.52, 1.09	0.130
45+	0.59	0.41, 0.87	0.007
Household size			
3 people or more	1.00		
1–2 people per room	0.73	0.56, 0.96	0.026
Residency			
Rural	1.00		
Peri-urban/urban	1.10	0.76, 1.63	0.594
Clinic of enrolment			
Clinic 1	1.00		
Clinic 2	3.02	2.12, 4.30	<0.001
Clinic 3	3.63	2.47, 5.33	<0.001
Multiple partners			
No	1.00		
Yes	3.01	0.98, 9.21	0.054
Missing	0.65	0.39, 1.08	0.097
Gel use			
Sometimes/never	1.00		
Always	1.50	1.12, 2.00	0.006
Discussed gel with partner			
Yes	1.00		
No	0.71	0.49, 1.03	0.069
Average number of sex acts			
1 to 5 acts	1.00		
6 + acts	1.48	1.09, 2.01	0.011

Since post-coital intravaginal cleansing would only affect the risk of HIV infection in the absence of condom use, we repeated the multivariate analysis after excluding sex acts where women reported using a condom. In this analysis, 22 % (249/1143) of women reported intravaginally cleansing less than 1 h after a sex act in which they did not use a condom. In general, the association between post-coital IVC and the independent variables seen in Table 4 did not change in the re-analysis. However, in the multivariate model the odds of intravaginal cleansing were significantly lower among women aged 25–34 (AOR 0.55, 95 % CI 0.36, 0.83) and 35–44 years old (AOR 0.52, 95 % CI 0.35, 0.78). Unlike in the earlier model, the associations between post-coital intravaginal cleansing and reporting multiple partners or discussing gel use with the partner were statistically significant (multiple partners AOR 3.68, 95 % CI 1.20, 11.34; discussed gel with partner AOR 0.60, 95 % CI 0.39, 0.92). On the other hand, the association with household size was no longer significant (AOR 0.81, 95 % CI 0.60, 1.10).

Of the 1,143 women included in our analysis, 1,065 provided data on intravaginal cleansing practices in both the first and second half of the trial. In the first half of the trial, from week 4 to week 24, 277 (26 %) women reported intravaginal cleansing less than 1 h after sex. In the second half of the trial, from week 28 to week 52, this had fallen to 138 (13 %). One hundred and ninety-eight (198) women reported intravaginal cleansing in the first half of the trial but not the second half. The only independent association with decreased cleansing was clinic of enrolment. With clinic one as the reference, women in clinic two were twice as likely to stop cleansing (OR 2.27; CI 1.51, 3.41) and women in clinic three were almost 3 times more likely to stop cleansing (OR 2.73; CI 1.82, 4.10) (data not presented due to space but available from authors on request).

### Qualitative FGD Analysis

#### Classification

Respondents agreed that intravaginal cleansing was a regular part of women’s general daily hygiene routine. In terms of vaginal cleansing after sex, respondents agreed that women used one of two practices, either external or internal cleansing. There were examples of both classification types provided in every FGD.

Approximately two-thirds of FGD respondents said women wiped outside their vagina after sex. Women reportedly wiped with a dry or damp cloth, towel, tissue or toilet paper. Some respondents even said that many women had a specific towel for this purpose that they kept at the head of their bed. This quote exemplifies a common theme regarding different cleansing practices (wiping externally versus washing internally) depending on whether a woman had sex in the day or in the night:

“If you have sex during the day you wash because you still have to go outside, so you cannot wipe with a towel. (At night) you wipe because you are going to sleep and you wash in the morning” (Community FGD, exact age unknown but one of seven respondents aged between 22 and 32 years old).

Approximately a third of FGD respondents said women washed inside their vagina after sex, even during the night. The respondents explained that women would usually get up after sex to go and wash. This was described as occurring immediately after sex, so within approximately an hour of sex. There were also frequent reports of women placing a basin of water next to the bed at night in order to wash after sex:

“I do not know what the other people do but with me I put my water next to me when I sleep so that immediately after sex I take it and wash myself because I hate the sperm” (Community FGD, 35-year-old woman).

Intravaginal cleansing involved the insertion of either cloth or fingers. Respondents described the use of fingers to clean intravaginally after sex in 4 out of 10 FGDs with trial participants and 2 out of 6 community FGDs with women. However, no one mentioned finger cleansing in any of the 11 community FGDs with men, suggesting that women practice this privately. In the majority of cases, intravaginal cleansing also included the use of water. Respondents reported that women used plain, usually cold, water. Only a few women and men mentioned the use of disinfectants (liquid Dettol or Savlon) in the water.

#### Motivation

Many women described the vagina as requiring specific cleaning because, as this woman explained:

“We were given a smelly piece of organ” (Community FGD, 59-year-old woman).

Respondents also described semen as being dirty and smelly. The combination of semen, vaginal discharge and sweat required that women clean themselves after sex. Respondents described the need to remove the smell of sexual fluids as a necessary part of having self-respect. One woman explained that women wash after sex “if one is a woman who loves herself” but also explained that:

“There are women who do not love themselves: she does not wash after sex even during the day. She would have a bad smell because sperm or discharge keeps on coming out” (Community FGD, 31-year-old woman).

Similarly, respondents frequently described intravaginal cleansing as a necessary part of respecting your partner and others in the household:

“The expectation is that the woman brings water in her bedroom so that she washes (intravaginally) first before meeting people and making tea for them” (Community FGD, 59 year old woman).

However, half a dozen respondents, a mix of female and male, suggested that it could be a sign of disrespect to a man for a woman to wash after sex:

“Some men will say don’t wash because it will lower their dignity” (Community FGD, 19 year old man).

Except for men’s lack of knowledge of finger cleansing, there were no obvious distinctions in opinion between women and men regarding how, when or why women intravaginally cleansed after sex. Women knew more than men about vaginal cleansing practices, but this was expected. Similarly, there were no obvious differences between group discussions with younger versus older people, or rural versus peri-urban respondents. There was clear agreement that it was necessary to clean after sex in order to remove both female and male sexual fluids.

There were a few unprompted conversations about the health implications and health benefits of intravaginal cleansing. In a community FGD, one woman stated that it was not necessary to wash after sex as women were not advised to do so at the primary health care centres. Respondents mentioned the idea of washing after sex to reduce the risk of HIV in 4 out of 11 community FGDs with men and 1 out of 6 community FGDs with women, but none with trial participants. Men raised this issue more than women, probably because the benefits of washing after sex were viewed as more pertinent to men, as this FGD exchange demonstrates:

“I heard another sister saying that after sex it is important to wash but using moving water like in the shower because if one does not wash after sex one might get HIV infection but washing immediately after sex helps to avoid HIV infection”(Community FGD, 26 year old woman).

“I heard that but I do not believe it. Maybe it is better for men but the (female) abdominal structure allows things to enter inside, so even washing will not help me” (Community FGD, 30 year old woman).

It was noteworthy that only one focus group discussed condom use in the context of intravaginal cleansing and stated that using a condom did not reduce the need to cleanse intravaginally.

### Qualitative IDI Analysis

In the in-depth interviews, women agreed that it was common to either wipe outside the vagina or wash inside the vagina immediately after sex. The motivations provided for intravaginal cleansing in the IDIs, mirrored those provided in the FGDs.

#### Intravaginal Cleansing Among Trial Participants

During the IDIs, women’s comprehension of many of the key trial messages was assessed—for example their understanding that the gel was investigational and that the gel could not be used when pregnant. However, we did not assess their understanding of the message not to intravaginally cleanse less than 1 h after sex thoroughly in the interviews. It was obvious that some women clearly understood this requirement, but often it was not clear whether women who continued to cleanse intravaginally less than 1 h after sex understood that this could potentially limit the effectiveness of a microbicide gel.

Of the 84 women interviewed, 33 reported intravaginally cleansing immediately after sex in at least one in-depth interview, although reports were highest at the week 4 interview declining by the week 24 and 52 interviews. Some women reported just using water to wash internally. However, no one reported using a douching device of any sort so it was not clear from the interviews how exactly women inserted water. A few women reported using soapy water, specifically referring to the use of ‘Sunlight’, which is a popular soap brand in South Africa. Some women reported just using face cloths or towels to clean intravaginally, usually dampened. Approximately half of the women reported inserting either a single finger or multiple fingers in order to clean inside the vagina after sex. Some reported just using their fingers to clean while other women reported using a cloth over the fingers:

“It is the towel which gets inside together with the finger too, though the finger is in the towel” (Trial IDI, 39-year-old woman).

In the IDIs, respondents regularly mentioned the impact of condom use on intravaginal cleansing. For the majority, the use of a condom did not alter their need to cleanse after sex as they still found it necessary to remove their own vaginal fluids. However, a few women reported that they were less inclined to cleanse intravaginally after sex if their partners had worn condoms:

“Before I started using condoms I used to wash…..Now there is no dirtiness because I am using condoms” (Trial IDI, 46 year old woman).

#### Microbicide Gel Use

Two women thought that they were *supposed* to clean after sex in order to remove the gel. Despite counselling not to intravaginally cleanse less than an hour after sex, some women continued to do so, as it was their usual practice. This woman refers to her ‘sperm’, which is a term commonly used to refer to female sexual fluids, as well as male, in this community:

“I like cleaning myself, so as to remove gel and my sperms (*ama*-*sperms*), because it’s not easy for his dirt to get into me because we would have used a condom. I just wash to clean my dirt” (Trial IDI, 39-year-old woman).

Other women specifically cleansed intravaginally between sex acts. This woman was asked why she intravaginally cleansed between sex:

“To remove the old gel because it will not work…. I insert fingers, wipe with a towel, and insert the gel….After we had sex I wash to remove the old gel because I don’t want him to want sex again before I have inserted the gel again” (Trial IDI, 46 year old woman).

Approximately half a dozen women explained that they used to wash intravaginally after sex, but since joining the trial and receiving counselling not to, no longer wash intravaginally if they used gel:

“I know that I should wash after having sex, but if I used the gel I don’t wash because it was said that I shouldn’t wash (internally), I should wipe (externally)” (Trial IDI, 26 year old woman).

Interestingly there were no reports of women merely delaying intravaginal cleansing for more than an hour after sex based on counselling from the research staff not to cleanse *within* an hour. There were no suggestions of why some women continued to intravaginally cleanse immediately after sex when using gel and others did not, except for women’s individual attitudes to, and preference for, post-coital cleansing.

#### Sex During Menstruation

Most respondents believed that sex during menstruation was rare, describing it as culturally and religiously unacceptable. In the IDIs, sex during menstruation was described as dirty (*ngcolile*), smelly (*nuka*), shameful (*amahloni*), disgraceful or disgusting (*ihlazo*), embarrassing (*ukuhlaziswa*) and as a sign of a lack of self-respect (*ukuzenyanya* – does not love oneself). Indeed, the Shembe^1^ religion [37], which was the second largest religion, expressly forbids women to have any contact with men during menses, as this quote illustrates:

“I don’t prepare him food when I am menstruating. Food for him is prepared by the children, I don’t even sleep in his bedroom, I leave his bedroom” (Trial IDI, 48-year-old woman).

Nonetheless, respondents suggested that women were more likely to cleanse intravaginally after sex during menstruation. In fact, a number of women reported that they only intravaginally cleansed after sex if they have sex whilst menstruating:

“You can usually wash only when you have been doing sex whilst menstruating, then you could maybe wash because of that reason” (Trial IDI, 33-year-old woman).

Having sex during menstruation was most frequently attributed to labour migration, whereby if the couple were only together for a short period of time when a migrant labourer was home and the women was menstruating the whole time, then they would not forego sex. Some respondents believed that the body was weak during menstruation and therefore more prone to infection during sex. Despite the objections to sex during menstruation, 14 of the 84 women reported having sex during menstruation while in the trial. Over half reported typically using gel when having sex during menstruation, and over half reported intravaginal cleansing after sex during menstruation.

## Discussion

In this study conducted in KwaZulu-Natal, South Africa, we found that the majority of women did not report post-coital intravaginal cleansing at any time during the microbicide trial. However, one-third of women reported doing so less than 1 h after sex, despite receiving counselling not to do so. Post-coital intravaginal cleansing is clearly an important practice for some women in terms of managing their sexual health and sexuality [19]. Nonetheless, our study suggests that this practice may be amenable to change. Nearly half the women who said they intravaginally cleansed during the first 6 months of the trial did not do so during the second 6 months after repeated counselling to refrain from intravaginally cleansing within an hour after sex.

The prevalence of post-coital intravaginal cleansing was higher in this community than previously reported in KwaZulu-Natal [13]. In this population, younger age, household crowding and increased sexual activity were associated with post-coital intravaginal cleansing, although condom use was not. Other studies in South Africa have found age to be associated with intravaginal cleansing generally, not just after sex [6, 7, 11]. In our study, women over 45 years of age were least likely to report post-coital intravaginal cleansing. In contrast to our findings, a household survey among 18–60 year old women found women aged 30–44 were significantly less likely to report any type of intravaginal practice than women in younger and older age groups [11]. The qualitative data did not help explain why these practices differ by age. However, other qualitative work in KwaZulu-Natal has suggested that women increase intravaginal cleansing practices when they are trying to attract a new partner, especially male partners younger than themselves [38]. We did not collect data on the age of partners and therefore were unable to test this hypothesis.

The quantitative findings demonstrated that women in larger households were more likely to cleanse intravaginally after sex. The qualitative data offers a possible explanation for this finding. Households in KwaZulu-Natal are often patrilineal and multi-generational, and there were frequent references to the need to be ‘clean’ before greeting other people in the household as a sign of respect. In contrast, household crowding was inversely associated with intravaginal cleansing in Madagascar [39]. The divergence of findings from South Africa and Madagascar highlights the impact of socio-cultural influences on intravaginal practices and illustrates the need to consider the impact of residential circumstances on intravaginal cleansing practices in different societies. This is of particular interest given recent evidence that household size also affects women’s use of microbicide gels in Uganda [40].

In this analysis, the prevalence of post coital intravaginal cleansing was higher among women who reported having sex more often. Other studies in Southern Africa have found an association between intravaginal cleansing and sexual activity [6, 7, 11, 39]. Our analysis is unusual in measuring the impact of sexual frequency on post-coital intravaginal cleansing among sexually active women who are not engaged in commercial sex work. The qualitative data also suggests a relationship between intravaginal cleansing and sexual frequency as some women purposefully cleansed intravaginally to remove the old gel in preparation to insert the new gel for the next act of sex. This finding regarding the association with sexual activity could have particular implications for microbicide dosing strategies that require peri-coital insertion.

Other studies in South Africa have found that intravaginal practices, although not specifically post-coital intravaginal cleansing, are reported less by women who use condoms [6, 9, 11, 41]. Van der Straten suggests that “the use of male condoms should prevent any post-coital discharge, and hence, this may in part explain lower vaginal practices” [42, p 597]. In contrast, our findings demonstrate that intravaginal cleansing is influenced equally by the need to remove vaginal sexual fluids and sweat, as well as semen. This is supported by substantial evidence that people define both semen and post-coital vaginal secretions as smelly, dirty and polluting in many parts of Africa [13, 22, 43–47].

The qualitative data suggests that intravaginal cleansing mainly involves water, fingers and/or a cloth. These forms of intravaginal cleansing have been dominant in previous studies [6, 11, 13, 22, 43, 48–55]. Unlike reports from other studies in KwaZulu-Natal [13], there were few reports in this analysis of commercial or other products being used for intravaginal cleansing. However, this is still cause for concern as intravaginal use of cloth or paper, as well as intravaginal cleansing with soap, and intravaginal insertion of products to dry or tighten the vagina, is associated with a significant increased risk of HIV acquisition [56].

A number of issues emerged during the analyses that require further attention in future studies. Firstly, it is of particular interest that intravaginal cleansing was more common among consistent compared to inconsistent gel users. The fact that we measured post-coital intravaginal cleansing during a year of follow-up, may explain why we recorded higher prevalence than the WHO survey in KwaZulu-Natal [13]. However, we cannot rule out the possibility that the use of gel influenced intravaginal cleansing. In another study where intravaginal cleansing was associated with using HEC placebo gel, compared to the less viscous Acidform gel, the authors concluded that: “gels may have been sensed as moisture or wetness, … as more gel accumulated in the vagina, women may have experienced a greater compulsion to cleanse despite having been instructed not to do so” [39, p 193]. In contrast a diaphragm trial found that in the intervention arm, women who intravaginally cleansed were less likely to report consistent use of a lubricant gel when administered in a diaphragm [9]. However, the authors attributed this more to the diaphragm than the presence of the gel, which has been shown elsewhere [10, 57]. In this analysis post coital intravaginal cleansing did not differ by gel group, although collectively these findings highlight the need to continue measuring intravaginal cleansing in relation to new microbicides formulations. While we measured the proportion of women who ever reported post-coital intravaginal cleansing, we did not calculate the frequency with which women cleansed. We plan to conduct a frequency analysis using the entire MDP 301 dataset which will be important in terms of considering the implications of post-coital intravaginal cleansing on the ability of trials to measure microbicide effectiveness.

Evidence from MDP 301 and other microbicide trials has shown that many women describe microbicides as being cleansing and hygienic [58–61]. Our findings demonstrate that we need to understand more about women’s perception of microbicides and their cleansing properties, as well as exploring the broader implications of this relationship between using vaginal microbicides and post-coital intravaginal cleansing. The suggestion that product use (microbicide or diaphragm) influences intravaginal cleansing practices, or vice versa, is critically important for the future of HIV prevention and requires far more focused attention in future research.

The second issue relates to intravaginal cleansing after sex during menstruation. Despite cultural taboos surrounding sex during menstruation [45], both the quantitative and qualitative findings confirm that a minority of women do have sex during menstruation in this community. In the quantitative analysis, ‘ever’ having sex during menstruation was not associated with intravaginal cleansing after sex, although we were unable to measure intravaginal cleansing specifically after sex during menstruation. Collectively the quantitative and qualitative analyses show that some women have sex during menstruation and use gel when having sex during menstruation. The qualitative findings suggest that during menstruation women may be more inclined to cleanse intravaginally after sex. Increased intravaginal cleansing during menstruation is well documented [13, 22, 41, 62]. However, there has been little attention to intravaginal cleansing specifically in relation to sex during menstruation. These findings highlight the need to understand more about sex during menstruation, gel use at time of sex during menstruation and intravaginal cleansing after sex during menstruation.

The third issue relates to behaviour change. Although the prevalence of intravaginal cleansing up to an hour after sex in this study was not optimal for microbicide use, it does appear from both the quantitative and qualitative data that some women were willing to stop intravaginal cleansing when using microbicides. The fact that some women misunderstood the messaging and assumed they *should* remove the gel after sex, illustrates the need for consistent counselling regarding intravaginal cleansing. Similarly, the fact that intravaginal cleansing practices differed between clinics, but not by area of residence, and declined over time differentially by clinic, suggests that the differences may relate to counselling messages. Counselling has been shown to decrease intravaginal practices among women in other microbicide and diaphragm trials, although in some studies this has had a bigger impact on reducing intravaginal insertion than cleansing [9, 29, 39, 63]. Counselling has been used successfully in the USA to bring about a reduction in intravaginal cleansing [64]. Counselling messages regarding the use of microbicides and intravaginal cleansing need to be developed and evaluated, and we need to ensure that the decrease in cleansing observed in this study was not an artefact of post-coital intravaginal cleansing merely being practiced inconsistently and in response to specific circumstances.

The main strength of this analysis is that it is the first to measure intravaginal cleansing less than 1 h after sex, which is the period of greatest relevance for microbicide gel use. However, one limitation of the quantitative analysis is that we rely solely on self-reported intravaginal cleansing data from the administered questionnaires. A previous study found that, compared to administered questionnaires, pictorial daily self-completed diaries can improve the accuracy of data on cleansing frequency and cleansing in proximity to sex [65, 66]. Interestingly, a study in Tanzania found that a higher proportion of women reported vaginal washing (although not specifically intravaginal cleansing) in face-to-face interviews compared with coital diaries, suggesting a social desirability bias towards over reporting washing practices [66]. We cannot rule out the fact that IVC was over or under-reported in this analysis, although the fact that the quantitative data are remarkably consistent with the qualitative IDI data, increases confidence in the estimated prevalence of post-coital intravaginal cleansing in this cohort. Other limitations of these analyses are that we did not explore why women stopped intravaginal cleansing and what impact this had on their overall vaginal hygiene practices, and whether women who continued post-coital intravaginal cleansing understood that this could potentially limit the effectiveness of a microbicide gel. Given no-one in this study reported the use of douching devices, we also missed an opportunity to explore the exact mechanisms by which women intravaginally cleansed with water alone.

We did not find any associations between intravaginal cleansing and educational level, employment type, contraceptive use, or HIV/STI prevalence, as has been observed in other studies in South Africa [6, 7, 11, 13, 39]. However, it is a limitation of this study that we were not able to test other factors that have been shown to be associated with intravaginal practices, including marital status, religiosity, concern about STIs, concern about partner’s fidelity, and access to media [6, 7, 11, 13].

## Conclusion

Although the majority of women in the Africa Centre MDP 301 microbicide trial in KwaZulu-Natal did not report intravaginal cleansing less than 1 h after sex, about one-third of women did report this practice despite repeated counselling to the contrary. Nonetheless, the analysis suggests that this practice may be amenable to change. In order to develop effective messages and counselling practices, it is vital that we understand more about the impact of post-coital intravaginal cleansing on product efficacy and explore further the association between gel use and post-coital intravaginal cleansing. If post-coital intravaginal cleansing significantly reduces the efficacy of microbicides, whether delivered before or after sex or in a vaginal ring, then cleansing practices could undermine the efficacy of microbicides for some women in the absence of effective behaviour change programmes.

## Footnotes

1 Shembe is based on the teachings of Isaiah Shembe (1867–1935), is part of the Nazareth Baptist Churches and is the oldest African Independent Church in South Africa, concentrated among Zulu populations. Shembe promotes traditionalist Zulu values and beliefs, such as polygamy, the role of the ancestors and the use of faith healing.
